# Evaluation of IndiMix JOE with Intype IC-RNA as an Alternative to AgPath-ID for Influenza A Virus Detection in Avian and Bovine Samples

**DOI:** 10.3390/pathogens15060600

**Published:** 2026-06-03

**Authors:** Andie Hach, Anne Vandenburg-Carroll, Douglas G. Marthaler, Stephen Vadia, Qirui Zhang, Melanie Prarat, Ailam Lim

**Affiliations:** 1 Wisconsin Veterinary Diagnostic Laboratory, University of Wisconsin–Madison, Madison, WI 53706, USA; ahach@wisc.edu (A.H.); vandenburg@wisc.edu (A.V.-C.); 2 INDICAL BIOSCIENCE, 04103 Leipzig, Germany; douglas.marthaler@indical.com; 3 Ohio Department of Agriculture, Animal Disease Diagnostic Laboratory, Reynoldsburg, OH 43068, USA; stephen.vadia@agri.ohio.gov (S.V.); qirui.zhang@agri.ohio.gov (Q.Z.); melanie.prarat@agri.ohio.gov (M.P.); 4 Department of Pathobiological Sciences, University of Wisconsin–Madison, Madison, WI 53706, USA

**Keywords:** influenza A virus surveillance, highly pathogenic avian influenza, exogeneous internal controls, diagnostic method comparison, limit of detection, analytical sensitivity, RT-PCR, PCR inhibition, outbreak response, IndiMix JOE, intype IC-RNA, AgPath-ID One-Step RT-PCR Reagents, bovine milk, bovine semen

## Abstract

This method comparison demonstrated equivalent analytical and diagnostic performance of IndiMix JOE with intype IC-RNA as an alternative real-time polymerase chain reaction (RT-PCR) chemistry compared to the National Animal Health Laboratory Network (NAHLN) influenza A virus (IAV) RT-PCR surveillance assay using AgPath-ID One-Step RT-PCR Reagents in avian swabs and tissues and bovine milk. In avian samples, IndiMix JOE with intype IC-RNA using a fast reduced-volume protocol had comparable results to the NAHLN reference RT-PCR with and without intype IC-RNA using standard NAHLN thermocycling conditions. A six-way comparison of RT-PCR chemistries and exogenous internal controls in milk samples illustrated equivalent mean CT values (ANOVA, *p* = 0.9938). Additional experiments in avian swabs and tissues, bovine milk and semen samples resulted in comparable analytical sensitivity in limits of detection (LOD), linearity (R^2^ > 0.977), and PCR efficiencies, and no significant differences in mean CT values (ANOVA, *p* > 0.05). Diagnostic performance showed 100% agreement across field and spiked sample matrices compared to the NAHLN reference method. The study supports the use of IndiMix JOE and intype IC-RNA as an alternative, with practical improvements relevant to surveillance workflows including enhanced testing flexibility, efficiency, outbreak response capacity, and reduced time to results.

## 1. Introduction

Highly pathogenic avian influenza (HPAI) A(H5N1) clade 2.3.4.4b has posed a major global animal health threat since late 2021. Between January 2022 and February 2026, the virus spread extensively across United States poultry, affecting over 2210 commercial and backyard flocks and resulting in the loss of more than 195 million birds across all 50 states and one territory [1]. The virus has circulated widely in wild birds through repeated introductions via migratory flyways, and spillover into mammals has been reported in multiple countries, underscoring its expanding host range and ecological impact [2,3,4,5,6,7,8]. In March 2024, the H5N1 B3.13 genotype was identified in U.S. dairy herds and spread rapidly to more than 1000 dairy operations within the first year [9,10,11]. Ongoing global surveillance of HPAI in cattle has identified emerging genotype D1.1 by the U.S. Department of Agriculture (USDA) National Milk Testing Strategy, while the detection of antibodies in dairy cattle in the Netherlands highlights the potential global impacts in livestock [12,13,14,15,16].

Detection of HPAI H5N1 in bovine biological materials raises questions about potential viral shedding in semen and implications for reproduction and herd health [17]. Semen, like milk, is a challenging matrix for molecular diagnostics due to the presence of inhibitors such as proteins, lipids, and other compounds that can interfere with nucleic acid extraction and PCR amplification [18,19,20,21]. These factors complicate reliable detection of viral RNA, necessitating optimized extraction protocols and exogenous internal controls (IC) to ensure assay sensitivity and specificity [18]. Understanding and overcoming these technical hurdles is critical for informed biosecurity measures, surveillance programs, and risk assessments of HPAI.

National Animal Health Laboratory Network (NAHLN) laboratories provide the backbone for HPAI surveillance in the U.S., using standardized real-time polymerase chain reaction (RT-PCR) assays for rapid detection in poultry, wild birds, and livestock [1,4,11]. Alternative RT-PCR chemistries and exogenous control detection strategies are essential for maintaining surveillance capacity, mitigating reagent constraints, and avoiding reliance on a single vendor. The use of multiple vendors offers competitive pricing, adds flexibility to workflows, and helps ensure continuity of testing. The COVID-19 pandemic exposed critical supply chain vulnerabilities, reinforcing the importance of diversified sourcing to sustain uninterrupted diagnostic operations [22]. Currently, two RT-PCR chemistries, AgPath-ID One-Step RT-PCR Reagents (AgPath-ID) and the VetMAX-Gold Avian Influenza Virus (AIV) Detection Kit, and a single exogenous control, VetMAX Xeno Internal Positive Control RNA (Xeno), supplied by Thermo Fisher Scientific (Waltham, MA, USA), are approved. Alternative detection strategies, including multiplex PCRs, positive internal controls (ICs), and improved extraction methods for complex matrices such as semen are being explored to enhance sensitivity and reliability [18,23].

The goal of this method comparison was to demonstrate the equivalence of Indical Bioscience’s (Leipzig, Germany) fast and reduced-volume (FRV) protocol using the IndiMix JOE master mix and intype IC-RNA (intype IC) across multiple sample matrices to AgPath-ID and Xeno. This alternate chemistry approach is intended to support and enhance IAV diagnostic testing capacity within NAHLN laboratories during surveillance and outbreak response, enabling timely outbreak management and regulatory decision-making.

## 2. Materials and Methods

### 2.1. Field and Reference Samples

Avian swabs and tissue, bovine milk and semen samples are routinely submitted to the Wisconsin Veterinary Diagnostic Laboratory (WVDL) for pathogen screening. The avian and semen samples were described in previous studies [18,24]. The avian and semen samples used for limit of detection (LOD) testing were spiked with three low-pathogenicity influenza A strains (LPAI) to assess analytical sensitivity, as described in previous studies. Negative milk samples were pooled to create stock for the serial dilutions of the three LPAI strains to determine the LOD. In sum, 53 avian field samples (20 positive and 33 negative) and 57 semen samples (27 spiked positive and 30 negative) from previous studies were used for diagnostic sensitivity and specificity testing [18,24]. For milk samples, diagnostic sensitivity and specificity were assessed using 100 previously positive field milk samples and 231 previously negative samples. The status of the previously described samples was determined using the NAHLN reference method, as described in the subsequent section.

Lastly, an inter-laboratory comparison was conducted using a proficiency panel of 16 inactivated milk samples produced by the Veterinary Laboratory Investigation and Response Network (Vet-LIRN). The known HPAI status and concentration was determined by Vet-LIRN. Samples were frozen in an ultralow freezer at −80 °C upon receipt and between studies.

### 2.2. Extraction Chemistries and Equipment

The avian samples (swabs and tissues) were extracted using 50 μL of sample input and 150 μL of 1× phosphate-buffered saline (PBS) with the various versions of the NAHLN-approved prefilled IndiMag Pathogen Kit (Indical Bioscience, Leipzig, Germany) on the IndiMag 48s, IndiMag 2 (Indical Bioscience, Leipzig, Germany), and the KingFisher Flex (Thermo Fisher Scientific, Waltham, MA, USA), as previously described [24]. Milk samples were extracted using 50 μL of sample input and 150 μL of 1× PBS with the prefilled IndiMag Pathogen Kit on the KingFisher Flex [25]. The semen samples were extracted using 100 μL of sample input and 100 μL of 1× PBS on the KingFisher Flex as previously described [18]. For all sample matrices, 1 µL of intype IC (Indical Bioscience, Leipzig, Germany) and 2 µL of Xeno (Thermo Fisher Scientific, Waltham, MA, USA) were spiked into the lysis buffers for all extractions. The extracted RNA was stored in an ultralow freezer at −80 °C between studies to minimize RNA degradation.

### 2.3. Real-Time Polymerase Chain Reactions (RT-PCRs)

The nucleic acid extracts were run on various RT-PCR assays for comparison. For avian samples, the NAHLN IAV protocol (NVSL-SOP-0068) was employed using the AgPath-ID (Thermo Fisher Scientific, Waltham, MA, USA) either without IC (AgPath-ID/without IC, NAHLN reference methods) [25] or with intype IC primers (forward and reverse at 160 nM each) and probe (fluorescent dye of JOE at 80 nM) (AgPath-ID/intype IC) at 25 µL reaction volume. The IndiMix JOE master mix (Indical Bioscience, Leipzig, Germany) contains the primers and probe to identify intype IC and was tested at 20 µL reaction volume using the NAHLN standard thermocycling parameters (IndiMix JOE/intype IC). In addition, IndiMix JOE was evaluated using a fast thermocycling protocol with a 20 µL reaction volume (IndiMix JOE FRV/intype IC). The thermocycling conditions consisted of one cycle of 50 °C for 10 min and 95 °C for 2 min and 40 cycles of 95 °C for 5 s and 57 °C for 30 s. The same concentration of IAV primers and probe was used in IndiMix JOE master mix as described in the NAHLN-approved protocols [25]. Comparison was performed as outlined by the NAHLN Methods Technical Working Group (MTWG).

For milk, a six-way LOD comparison of various RT-PCR chemistries and internal control combinations was conducted at the request of the USDA IAV reference laboratory. The comparison consisted of the NAHLN reference method for milk per NVSL-SOP-0068 using AgPath-ID with Xeno (AgPath-ID/Xeno) [25], AgPath-ID/without IC, AgPath-ID/intype IC, IndiMix JOE FRV/intype IC, IndiMix JOE with Xeno Internal Positive Control—LIZ Assay (Thermo Fisher Scientific, Waltham, MA, USA) (IndiMix JOE FRV/Xeno (LIZ)), and an IndiMix TAMRA master mix (Indical Bioscience, Leipzig, Germany) with Xeno assay (IndiMix TAMRA FRV/Xeno). Xeno (LIZ) was used with IndiMix JOE instead of Xeno, and IndiMix TAMRA was used with Xeno instead of IndiMix JOE to avoid competing signals in the same fluorescent channel in the thermocycler. The Xeno and Xeno (LIZ) assays were used at 0.8 µL per reaction. IndiMix TAMRA contains the primers and probe to identify intype IC. All AgPath-ID assays were tested at 25 µL reaction volume under the NAHLN standard thermocycling protocol, while fast thermocycling with a 20 µL reaction volume (FRV) protocol was used in all IndiMix assays. Analytical and diagnostic performance for milk and semen extracts was evaluated using the NAHLN reference method assay (AgPath-ID/Xeno) and the IndiMix JOE FRV/intype IC assay. 

The RT-PCRs were performed on the ABI 7500 or QuantStudio 5 (Thermo Fisher Scientific, Waltham, MA, USA) following the NAHLN-approved parameters or the FRV protocol [25]. The IAV, Xeno, and intype IC targets were analyzed at 5% of the maximum amplitude of their positive amplification control to account for any variation between RT-PCR runs. The 5% was selected as it represented the midpoint of the amplification curve. The NAHLN-approved protocols are managed by the NAHLN program office and may be requested by contacting nvsl.mastercontrol@usda.gov.

### 2.4. Analytical and Diagnostic Performances

Standard curves were generated using tenfold-diluted materials for each matrix. The endpoint dilution of the reference strains determined the limit of detection (LOD). The PCR efficiency and the coefficient of determination (R^2^) were obtained from the standard curve generated by the software.

The intra- and inter-assay precision for avian samples was assessed by running both high and low concentrations of the reference strain. The low concentration was prepared at approximately ten times higher than the concentration of the reported LOD endpoint, and the high concentration was a dilution with a 25- to 30-cycle threshold (CT) range. Each sample was extracted and subjected to RT-PCR in 5 replicates for 6 different runs. Diagnostic performances were evaluated using known positive and negative samples previously determined by the NAHLN reference methods. Testing on a subset of samples for all matrices was repeated by different technicians at WVDL on different days using different thermocyclers to evaluate intra-laboratory repeatability. An inter-laboratory comparison was conducted at WVDL and the Ohio Department of Agriculture, Animal Disease Diagnostic Laboratory (ODA) with the same panel, protocols and similar equipment.

### 2.5. Statistical Analysis

For statistical analysis, samples lacking IAV or IC amplification were assigned a CT value of 40, corresponding to the maximum number of assay cycles. Assay performance was assessed by determining the LOD (requiring at least two of the three replicates to have amplification), coefficient of determination (R^2^), PCR efficiency, and diagnostic sensitivity and specificity. Precision was evaluated using the standard deviation and the percentage coefficient of variation (CV). Correlation of repeated samples for intra-and inter-laboratory comparison was assessed using the Pearson coefficient correlation value (r). Statistical differences between methods were examined using either a *T*-test or an ANOVA test. A *T*-test was used to assess differences between a single evaluated method and the NAHLN reference method, while an ANOVA test was applied when multiple methods were evaluated. All statistical analyses were performed in GraphPad Prism version 10.6.0 (GraphPad, Boston, MA, USA), and figures were created with Tableau, public edition (Salesforce, San Francisco, CA, USA).

## 3. Results

Initially, this study aimed to compare IndiMix JOE with AgPath-ID without IC (NAHLN reference method), thereby demonstrating IndiMix JOE as an alternative RT-PCR chemistry for IAV NAHLN surveillance in avian samples. The intype IC (an exogenous internal control) was included to monitor inhibition and to prepare for a possible change in the requirement for an exogenous internal control for NAHLN avian species IAV surveillance. In the avian study, the IndiMix JOE fast thermocycling condition using a reduced 20 µL volume (FRV) protocol was investigated to decrease RT-PCR thermocycling time and reduce assay cost by using a lower assay volume.

### 3.1. LOD and Precision of Avian Samples

**Figure A1:**
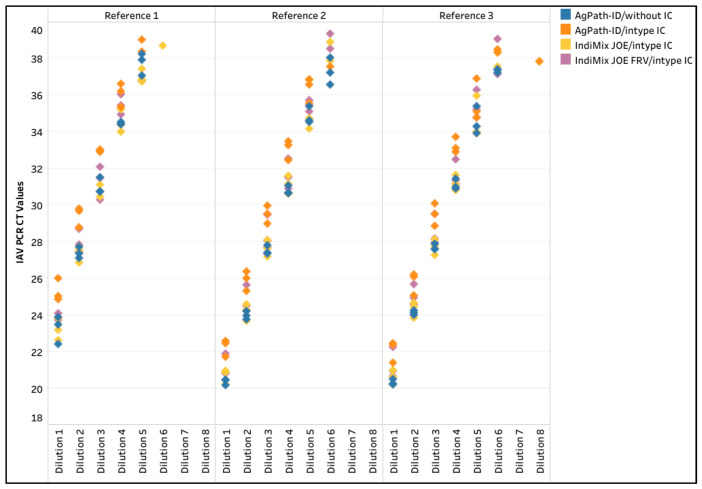
Influenza A virus (IAV) CT values across dilutions of three reference strains for AgPath-ID/without IC (blue), AgPath-ID/intype IC (orange), IndiMix JOE/intype IC (yellow), and IndiMix JOE fast, reduced volume (FRV) with intype IC (purple) in avian samples.

The three IAV reference strains tested in triplicate had comparable results across four protocols: AgPath-ID/without IC, AgPath-ID/intype IC, IndiMix JOE/intype IC, and IndiMix JOE FRV/intype IC (Table 1, Figure A1 and Supplemental Data S1). A minor reduction of one dilution level in LOD occurred with AgPath-ID/intype IC in one of the three reference strains. The IndiMix JOE FRV/intype IC had a reduced LOD of one dilution in sensitivity in one of the three reference strains. No significant difference was observed in mean CT values among the four RT-PCR protocols (ANOVA, *p* = 0.4597). The R^2^ values were highly similar (0.977 to 1.000) across the four RT-PCR protocols. Greater variability was observed in percentage PCR efficiencies within replicates and across the various RT-PCR protocols.

**Figure 1:**
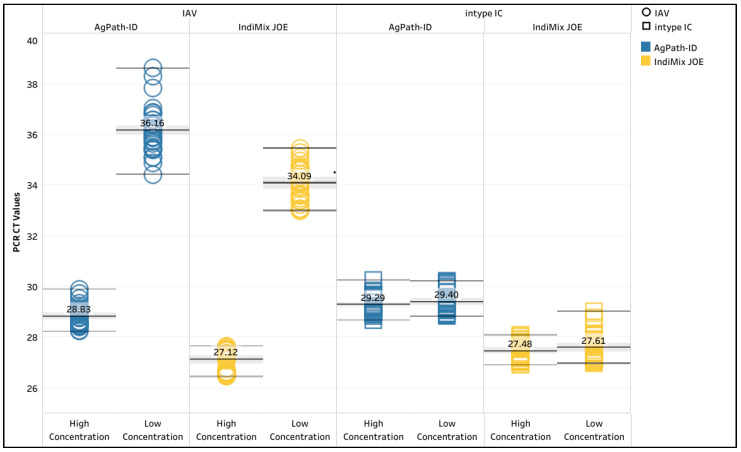
Precision of the high and low concentrations of influenza A virus (IAV, circles) and intype IC (squares) for avian samples using AgPath-ID (blue) and indiMix JOE (yellow). The mean CT value is shown, with the gray shaded area representing the 95% confidence intervals. The lower and upper lines represent the minimum and maximum CT values.

Assay precision was excellent and had low variability, with coefficients of variations (CVs) below the 3% acceptance threshold for both AgPath-ID/intype IC and IndiMix JOE/intype IC protocols (Figure 1 and Supplementary Data S2). The CVs for AgPath-ID were 1.43% and 2.61% at high and low IAV concentrations, respectively, whereas CVs of 1.56% and 2.09% were observed for the IndiMix JOE assay at high and low IAV concentrations, respectively. A similar trend was observed for the intype IC, with low CV ranging from 1.27% to 1.55% and 1.28% to 2.03% for the AgPath-ID and IndiMix JOE, respectively.

Additional experiments for diagnostic sensitivity and specificity, repeatability, and reproducibility were conducted in the avian study and are reported later with the milk and semen matrices.

### 3.2. AgPath-ID and IndiMix JOE with Xeno and Intype IC Comparison in Milk Samples

After the IndiMix JOE validation for NAHLN IAV avian surveillance was completed, HPAI was identified in dairy cattle in the U.S., and NAHLN IAV surveillance in milk required the addition of Xeno with the AgPath-ID assay. The USDA IAV reference laboratory requested a six-way comparison of the two exogenous internal controls in both RT-PCR chemistries.

**Figure A2:**
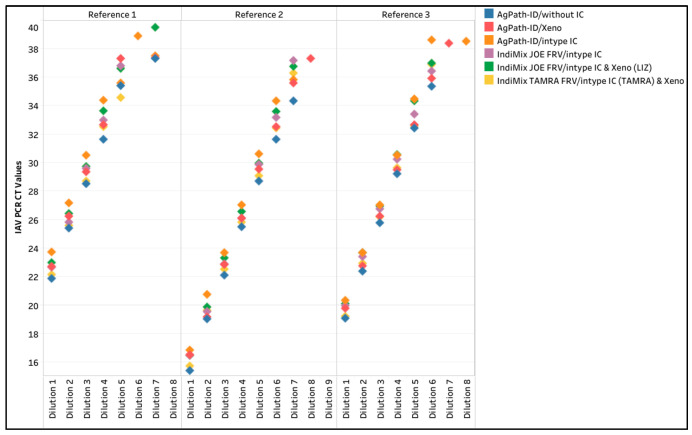
Influenza A virus CT values across dilutions of three reference strains for AgPath-ID/without IC (blue), AgPath-ID/intype IC (orange), IndiMix JOE FRV/intype IC (purple), IndiMix JOE FRV/Xeno (LIZ) (green), and IndiMix TAMRA FRV/Xeno (yellow) compared to AgPath-ID/Xeno (NAHLN reference method, red).

The overall LOD was consistent using a single replicate across the IAV reference strains for the six RT-PCR protocols (Table 2, Figure A2 and Supplemental Data S3). Compared to the NAHLN reference method (AgPath-ID/Xeno), a single-log reduction in LOD occurred for references 2 and 3 for all protocols. However, reference 1 showed a single-log increase in sensitivity when intype IC was added to AgPath-ID. The R^2^ values consistently ranged between 0.977 and 1.000 regardless of exogenous internal control or RT-PCR chemistry, but an increased fluctuation occurred in the percentage PCR efficiency, ranging between 90.2 and 133.7. The exogenous controls were within the NAHLN’s acceptable range (<34.5).

**Figure 2:**
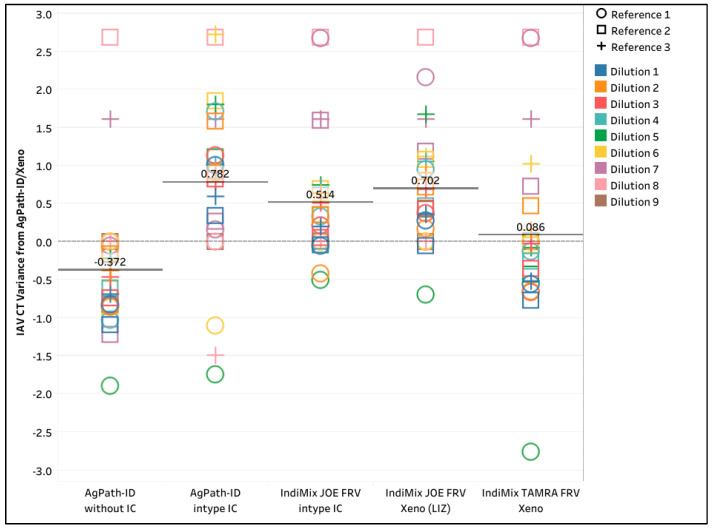
Influenza A virus CT variation across serial dilutions of three reference strains. Colors indicate dilution levels, and symbols (circle, square, and plus) represent individual reference strain for AgPath-ID/without IC, AgPath-ID/intype IC, IndiMix JOE FRV/intype IC, IndiMix JOE FRV/Xeno (LIZ), and IndiMix TAMRA FRV/Xeno compared to AgPath-ID/Xeno as the NAHLN reference method in milk. Negative variances indicate better performance (lower CT), while positive variances indicate higher mean CT compared to the NAHLN reference method.

While the LOD differed slightly from the NAHLN reference method (AgPath-ID/Xeno), no significant difference (ANOVA, *p* = 0.9938) occurred in the mean CT values across all six RT-PCR protocols. The variance in IAV CT values from the RT-PCR protocols was compared with the NAHLN reference method, and the mean variance ranged from −0.78 to 0.37, a normal, acceptable range for sample repeatability (Figure 2). The AgPath-ID/without IC had a lower mean variance by 0.37, while the IndiMix TAMRA FRV/Xeno assay was very comparable to the NAHLN reference method (only 0.09 mean CT higher). While not significant, the AgPath-ID/intype IC, the IndiMix JOE FRV/intype IC, and the IndiMix JOE FRV/Xeno (LIZ) had higher variances (0.51 to 0.78), but within a normal, acceptable range.

### 3.3. LOD in Milk and Semen Samples

After establishing performance equivalence between exogenous internal controls and RT-PCR protocols, extracted nucleic acid from extended semen from a previous study was added to the IndiMix JOE FRV/intype IC evaluation as a potential new sample type for IAV surveillance of bulls [17,18]. The results from milk and semen are presented together in the following paragraphs.

**Figure A3:**
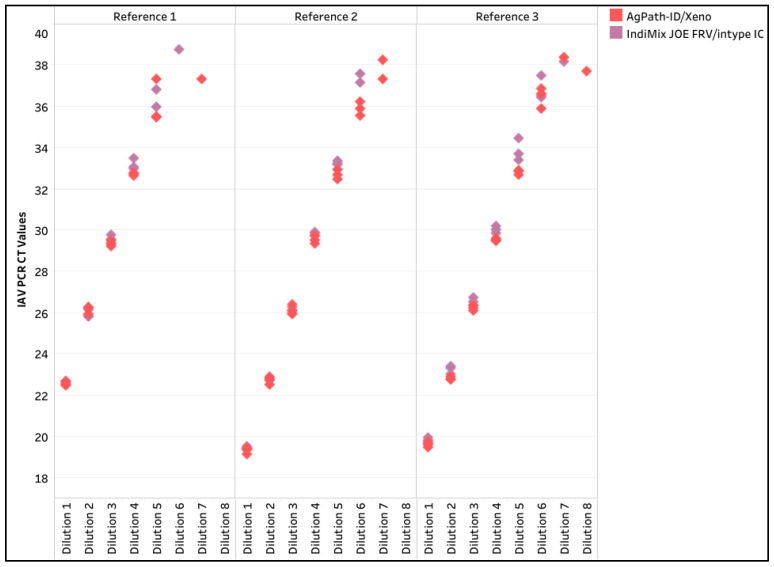
Influenza A virus (IAV) CT values across dilutions of three reference strains for the AgPath-ID/Xeno (red) and the IndiMix JOE fast, reduced volume (FRV) with intype IC (purple) in milk samples.

**Figure A4:**
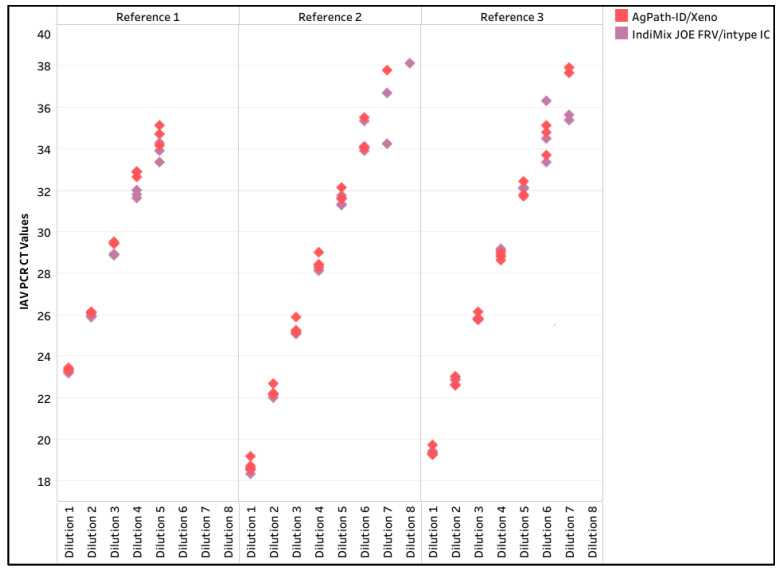
The influenza A virus (IAV) CT values across dilutions of three reference strains for AgPath-ID/Xeno (red) and the IndiMix JOE fast, reduced volume (FRV) with intype IC (purple) in semen samples.

The three IAV reference strains were tested in triplicate (similar to the avian study) with the NAHLN reference method (AgPath-ID/Xeno) and IndiMix JOE FRV/intype IC (Table 3, Figure A3 and Figure A4, and Supplemental Data S4). Compared to the NAHLN reference method, the LOD for IndiMix JOE FRV/intype IC was the same for milk and showed a single dilution increase in sensitivity for reference strain 2 in semen. The R^2^ values were excellent (≥0.99) across the various RT-PCR protocols and the two sample matrices. The variability in percentage PCR efficiencies was higher than expected, but highly similar when comparing the two RT-PCR protocols. For milk, the NAHLN reference method ranged from 90.2 to 106.2 compared to 89.3 to 101.9 with IndiMix JOE FRV/intype IC. For semen, the NAHLN reference method ranged from 98.5 to 120.7 compared to 98.5 to 118.9 with IndiMix JOE FRV/intype IC. Similar mean CT values were observed for IndiMix JOE FRV/intype IC compared with the NAHLN reference method in milk and semen matrices (*T*-test, *p* = 0.7011 and *p* = 0.7578, respectively).

### 3.4. Exogenous Internal Control Comparison from the Previous Triplicate LOD RT-PCR Methods with Avian, Milk, and Semen Samples

**Figure 3:**
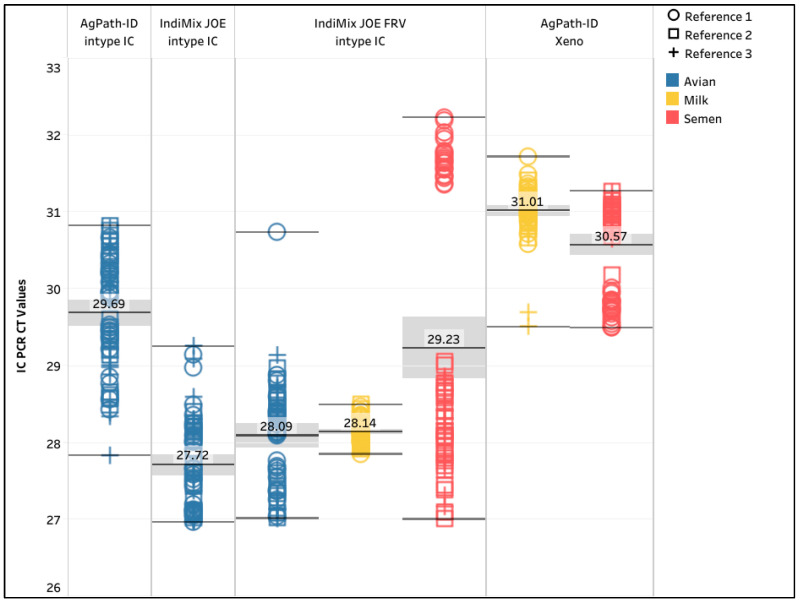
Intype IC and Xeno CT values in AgPath-ID and IndiMix JOE assays for avian (blue circles, squares, and pluses), milk (yellow circles, squares, and pluses), and semen (red circles, squares, and pluses) samples from the limit of detection section. The mean CT value is illustrated, with the gray shaded area representing the lower and upper 95% confidence interval. The lower and upper lines represent the minimum and maximum CT values.

The exogenous internal controls from the triplicate LOD from avian, milk, and semen samples (Table 1 and Table 3) were evaluated using the NAHLN acceptance CT cut-off value of 34.5 (Figure 3 and Supplemental Data S5). The mean intype IC CT values for AgPath-ID, IndiMix JOE, and IndiMix JOE FRV in the avian samples were 29.69, 27.72, and 28.09, respectively, with a significant difference (ANOVA, *p* ≤ 0.0001) between the AgPath-ID and IndiMix JOE assays (Figure 3). Statistical analysis was unavailable for milk and semen, as replicates of each IC in the matrices were lacking. The mean intype IC CT values with IndiMix JOE FRV were 28.14 and 29.23 in milk and semen samples, respectively. The CV for intype IC ranged from 2.15% to 2.48% for avian and 0.5% and 5.85% for milk and semen, respectively. A significant CT shift in the spiked reference strain 1, indicating inhibitory effects in the extended semen sample, contributed to the high CV in semen (Figure 3). The mean Xeno CT values were 31.01 and 30.57 in milk and semen samples, respectively. The CVs for Xeno were 1.00% and 1.88% in milk and semen samples, respectively.

### 3.5. Diagnostic Performance in Avian, Milk, and Semen

Next, the diagnostic performance of IndiMix JOE/intype IC and AgPath-ID/intype IC was compared to the NAHLN reference method (AgPath-ID/without IC for avian samples, and AgPath-ID/Xeno for milk samples). The number of positive and negative samples varied by sample type given the availability of sample matrices. There were 53 field avian samples (20 positive and 33 negative), 331 field milk samples (100 positive and 231 negative), and 57 semen samples (27 spiked positive and 30 negative) (Supplemental Data S6).

**Figure A5:**
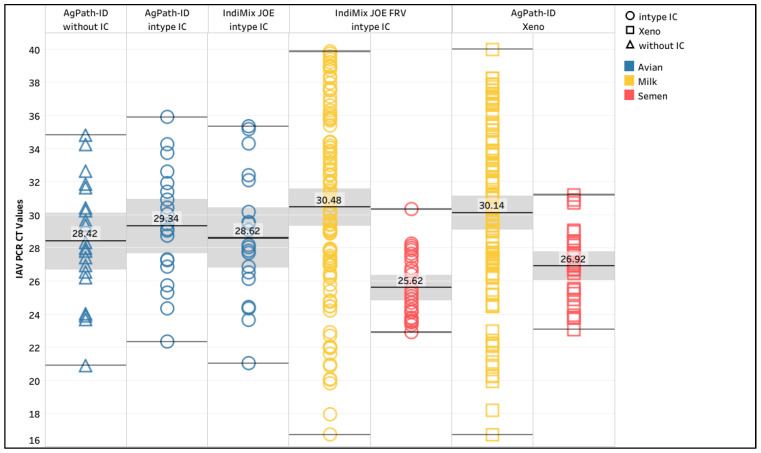
Influenza A virus (IAV) CT values in AgPath-ID and IndiMix JOE for positive avian (blue triangles and circles), milk (yellow circles and squares), and semen (red circles and squares) samples. The mean CT value is illustrated, with the gray shaded area representing the lower and upper 95% confidence interval. The lower and upper lines represent the minimum and maximum CT values.

AgPath-ID/intype IC and IndiMix JOE/intype IC for the avian samples and IndiMix JOE FRV/intype IC for the milk and semen samples had 100% agreement in diagnostic performance across the three sample matrices using field and spiked samples compared to the NAHLN reference method assays. The mean IAV CT values for the positive avian samples were 28.42 (95% CI: 26.71–30.14), 29.34 (95% CI: 27.70–30.97), and 28.62 (95% CI: 26.81–30.43) for AgPath-ID/without IC, AgPath-ID/intype IC, and IndiMix JOE/intype IC, respectively. The mean IAV CT values for the positive milk samples were 30.14 (95% CI: 29.10–31.18) and 30.48 (95% CI: 29.35–31.61) for AgPath-ID/Xeno and IndiMix JOE FRV/intype IC, respectively. The mean IAV CT values for the positive semen samples were 26.92 (95% CI: 26.04–27.79) and 25.62 (95% CI:24.84–26.39) for AgPath-ID/Xeno and IndiMix JOE FRV/intype IC, respectively (Figure A5). Significance was lacking between the mean IAV CT values for the RT-PCR protocols (avian, ANOVA *p* = 0.7181, and milk *T*-test, *p* = 0.1094). However, the mean IAV CT for semen by IndiMix JOE FRV/intype IC was significantly lower than by AgPath-ID/Xeno (*T*-test, *p* = 0.0265).

**Figure 4:**
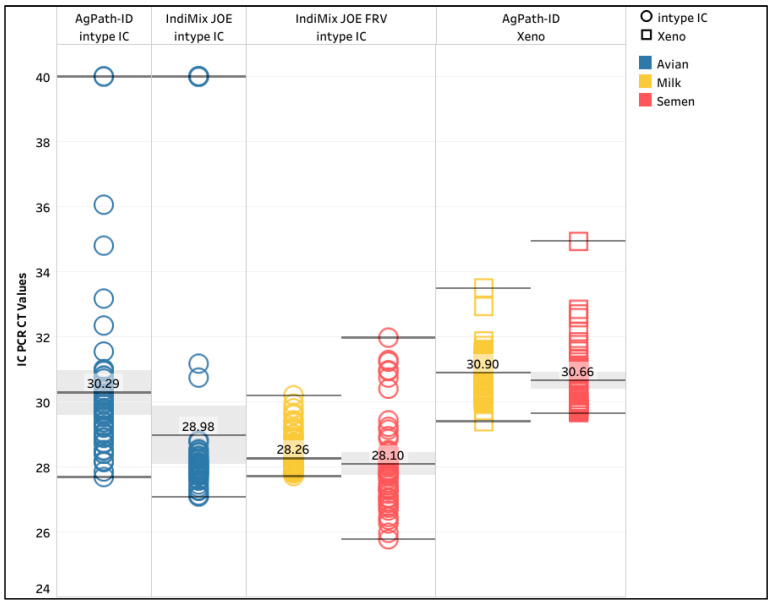
Intype IC (circles) and Xeno (squares) CT values in AgPath-ID and IndiMix JOE for avian (blue), milk (yellow), and semen (red) samples from the diagnostic performance section. The mean CT value is illustrated, with the gray shaded area representing the lower and upper 95% confidence intervals. The lower and upper lines represent the minimum and maximum CT values.

The exogenous internal controls in the positive and negative diagnostic samples were investigated using the NAHLN acceptance CT cut-off value of 34.5 (Figure 4 and Supplemental Data S7). The mean intype IC CT values were 30.29 (95% CI: 29.61–30.97), 28.98 (95% CI: 28.08–29.87), 28.26 (95% CI: 28.22–28.30), and 28.10 (95% CI: 27.73–28.46) for the various RT-PCR assays. The mean Xeno CT values were 30.90 (95% CI: 30.85–30.95) and 30.66 (95% CI: 30.39–30.92) for milk and semen, respectively. A significant CT shift in the intype IC was observed in two negative and two positive avian tissue samples, indicating inhibitory effects in those sample matrices (Supplementary Data S7).

### 3.6. Assay Repeatability and Reproducibility

**Figure 5:**
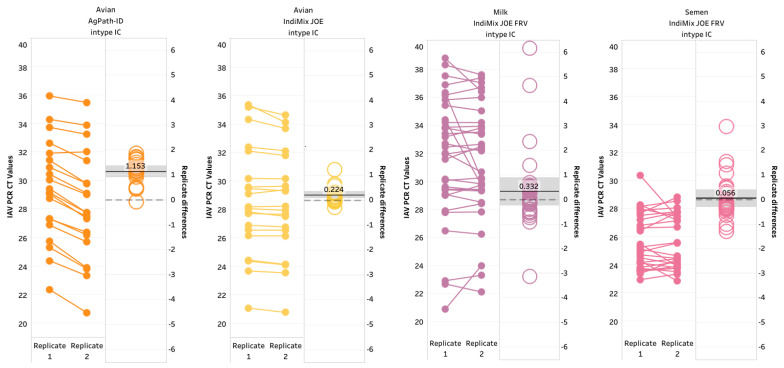
Intra-laboratory repeatability for avian (orange and yellow), milk (purple), and semen (pink) samples. The open circles represent the difference in CT values between replicates, with the mean variance and 95% confidence interval shown by the gray-shaded area. Pearson correlation coefficients r = 0.9944, r = 0.9974, r = 0.9351, and r = 0.8921 for the avian (AgPath-ID), avian (IndiMix JOE), milk, and semen datasets, respectively.

The intra-laboratory repeatability and reproducibility were investigated using 53 field avian samples (20 positives and 33 negatives), 32 field positive milk samples, and 57 semen samples (27 spiked-IAV positives and 30 negatives) (Supplemental Data S6). The positive and negative results were 100% in agreement with cross-classification analysis. The mean variance between replicates ranged from 0.056 to 1.153, indicating excellent correlations for the positive avian (AgPath-ID and IndiMix JOE), milk, and semen samples, with Pearson correlation coefficients, r = 0.9944, r = 0.9974, r = 0.9351, and r = 0.8921, respectively. (Figure 5). There was no significant difference between the replicates (*T* test, *p* = 0.3366, *p* = 0.8456, *p* = 0.7569 and *p* = 0.9164) for avian (AgPath-ID and IndiMix JOE), milk, and semen, respectively.

Next, variability in intype IC with the intra-laboratory repeatability and reproducibility dataset was investigated (Supplemental Data S7). With the replicates, the mean intype IC CT values were 29.35 (95% CI: 28.61–30.09), 29.42, (95% CI: 28.55–30.28), 28.29 (95% CI: 28.00–28.59), and 28.22 (95% CI: 27.89–28.54) for AgPath-ID and IndiMix JOE with avian, and IndiMix JOE FRV with milk and semen samples, respectively. The variances in the mean intype IC CT values were −0.94 (AgPath-ID, avian), 0.44 (IndiMix JOE, avian), 0.16 (IndiMix JOE FRV, milk), and 0.12 (IndiMix JOE FRV, semen), indicating excellent assay repeatability and reproducibility.

### 3.7. Inter-Laboratory Comparison

**Figure 6:**
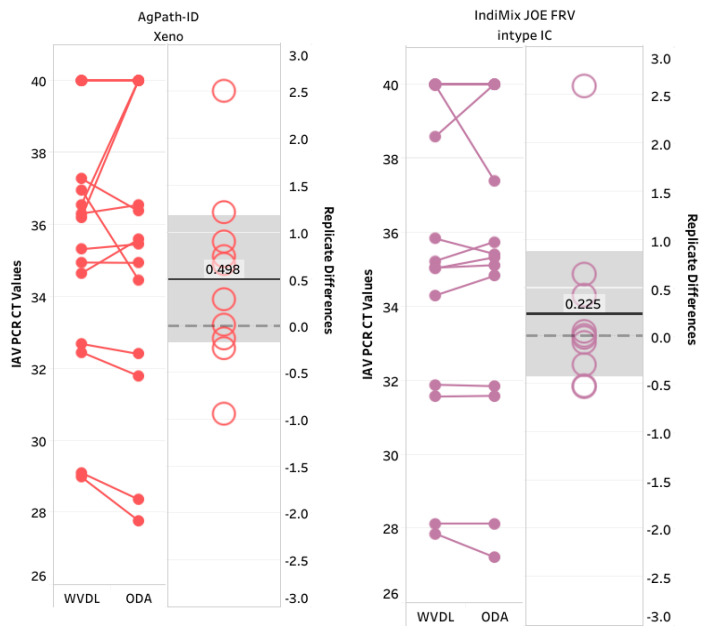
Inter-laboratory comparison of milk samples with AgPath-ID/Xeno (red) and IndiMix JOE FRV/intype IC (purple) at the Wisconsin Veterinary Diagnostic Laboratory (WVDL) and the Ohio Department of Agriculture, Animal Disease Diagnostic Laboratory (ODA). The mean CT value is illustrated, with the gray shaded area representing the lower and upper 95% confidence interval. Pearson correlation coefficients r = 0.9552 and r = 0.9702 for the AgPath-ID/Xeno and IndiMix JOE FRV/intype IC datasets, respectively.

Lastly, a proficiency panel of sixteen inactivated milk samples was tested by WVDL and the ODA with the NAHLN reference method (AgPath-ID/Xeno) and IndiMix JOE FRV/intype IC (Supplemental Data S8). The repeatable LOD (detection in all replicates) was 284 copies per 50 µL of milk for both the NAHLN reference method (CT values from 34.46 to 36.96) and IndiMix JOE FRV/intype (CT values from 34.29 to 35.83). Both RT-PCR protocols detected samples at 28 copies per 50 µL of milk in two of the three replicates (CT values > 36.38). The mean variances between replicates were 0.498 and 0.225, illustrating excellent correlations, with Pearson correlation coefficients r = 0.9552 and r = 0.9702 for the CT values of positive samples using the NAHLN reference method (AgPath-ID/Xeno) and IndiMix JOE FRV/intype IC, respectively (Figure 6). The mean Xeno CT values for individual comparisons were 30.84 and 29.30, while the mean intype IC CT values were 28.59 and 28.50 for WVDL and ODA, respectively.

## 4. Discussion

The NAHLN reference method used in this study is a robust IAV matrix assay that has been extensively utilized for routine avian influenza surveillance and across multiple HPAI outbreaks in the U.S. over the past two decades [26,27]. The assay updates over the years primarily focused on subtype-specific targets while maintaining the core matrix gene detection approach and a limited set of reagents. As an NAHLN-regulated assay, any addition of new reagents requires method comparison and approval by the NAHLN MTWG. Hence, this study followed a method comparison approach against the reference method as outlined by the NAHLN MTWG. Overall, the IndiMix JOE/intype IC results were highly comparable to the NAHLN reference methods across the different sample matrices. While variability in PCR efficiency was observed across RT-PCR methods, performance similarities were observed in mean CT values at the limit of detection (LOD) with excellent precision (CV < 3%) for both IAV and intype IC targets (CV 1.56–2.06% for IAV; 1.28–2.03% for intype IC), confirming the robustness of IndiMix JOE/intype IC within tested conditions. IndiMix JOE with the NAHLN reference thermocycling parameters was accepted by the NAHLN MTWG as an alternative method for IAV surveillance in avian species. The use of IndiMix JOE with the same thermocycling parameters as the NAHLN assays gives NAHLN laboratories additional flexibility to use the IndiMix JOE, thereby making the master mix interchangeable without workflow modifications.

Evaluation of avian clinical samples with known inhibitory effects (Supplementary Data S7) highlights the importance of incorporating an exogenous internal control to monitor PCR inhibition and prevent false-negative results, particularly in complex matrices rich in proteins and other inhibitory substances, such as tissues, milk, and semen [18,21]. The MIQE guidelines further emphasize the necessity of internal controls to ensure assay validity and reliability in diagnostic assays [28], and the World Organisation for Animal Health indicates an internal control must be included with inhibitor matrices to avoid a false-negative result [29]. Hence, Xeno was added to the NAHLN reference method when IAV was identified in milk [25]. The six-way comparison of RT-PCR chemistries and internal control configurations demonstrated no significant differences in mean CT values (ANOVA, *p* = 0.9938), and our comparison of intype IC and Xeno in milk demonstrated equivalent performance (ANOVA, *p* = 0.9938), supporting flexibility in internal control configuration while maintaining analytical integrity. Similar performance across internal control systems reinforces quality assurance principles in molecular diagnostics, and it provides maximum flexibility during high-demand outbreak situations or supply chain disruptions, which is particularly important and was experienced during the COVID-19 pandemic [22].

IndiMix JOE was also evaluated using a manufacturer-recommended, fast, reduced-volume protocol (IndiMix JOE FRV protocol) in all three matrices. The performance was highly comparable to that of the NAHLN reference method (AgPath-ID assays) under standard NAHLN thermocycling conditions. The approximately 30 min reduction in thermocycling time and a 20% decrease in reagent consumption translate into reduced time to result and lower reagent usage, providing meaningful operational and economic advantages for the NAHLN laboratories and agricultural industries [30,31]. The NAHLN MTWG approved this methodology for avian surveillance based on a large dataset, while approval for milk IAV testing is pending. Semen is currently not a sample matrix for NAHLN IAV testing given the lack of evidence of HPAI in semen, thus necessitating testing and the use of spiked IAV semen samples in this study.

Ideally, naturally infected samples would be used throughout the study. The nature of conducting research on HPAI samples negated this possibility, as well as an extensive interlaboratory comparison. Optimally, the study should have been conducted over a shorter period. Ultimately, the ongoing IAV outbreaks in U.S. poultry since February 2022 and the later detection of HPAI in lactating cattle in March 2024 have substantially increased demand for IAV surveillance and outbreak testing, which paused the study as the NAHLN program office and the laboratories tackled the high-volume testing for the outbreak [1,7,11].

## 5. Conclusions

Overall, the results across diverse sample matrices demonstrate that IndiMix JOE and intype IC, as alternative reagents, enhance the resilience and continuity of IAV testing within the NAHLN framework. Faster and alternative RT-PCR chemistries enhance diagnostic capacity by improving turnaround time, increasing workflow flexibility, and reducing reliance on a single reagent supplier, thereby supporting uninterrupted laboratory operations. Furthermore, the availability of interchangeable, performance-verified reagents ensures continuity of diagnostic capacity during outbreak response; mitigates risks associated with supply chain disruptions and supports rapid, reliable decision-making in animal health emergencies.

## Figures and Tables

**Table 1:** Influenza A virus (IAV) limit of detection (LOD), coefficient of determination (R^2^), and percentage PCR efficiency for AgPath-ID/without IC, AgPath-ID/intype IC, IndiMix JOE/intype IC, and IndiMix JOE fast, reduced volume (FRV)/intype IC in avian samples.

RT-PCR Chemistry		AgPath-ID	AgPath-ID	IndiMix JOE	IndiMix JOE FRV
Internal Control		without IC	intype IC	intype IC	intype IC
Category	Reference Strain	Avian			
LOD dilution level (2 of 3)	1	5	5	5	4
	2	6	5	6	6
	3	6	6	6	6
R^2^ value (range)	1	0.995–0.998	0.986–1.000	0.993–0.998	0.979–1.0000
	2	0.993–1.000	0.977–0.998	0.993–0.998	0.998–1.000
	3	0.993–0.998	0.992–1.000	0.999–1.00	0.997–0.999
Percentage PCR efficiency (range)	1	82.5–93.3	80.9–102.1	75.6–82.6	85.8–96.4
	2	93.0–97.6	82.7–108.8	81.1–92.8	90.3–95.8
	3	92.5–96.8	80.1–92.2	90.9–94.1	89.6–99.4

**Table 2:** Influenza A virus (IAV) limit of detection (LOD), coefficient of determination (R^2^), and percentage PCR efficiency for AgPath-ID/Xeno as the NAHLN reference method, AgPath-ID/without IC, AgPath-ID/intype IC, IndiMix JOE FRV/intype IC, IndiMix JOE FRV/Xeno (LIZ), and IndiMix TAMRA FRV/Xeno in milk.

RT-PCR Chemistry		AgPath-ID			IndiMix JOE FRV		IndiMix TAMRA FRV
Internal Control (IC)		without IC	Xeno	intype IC	intype IC	intype IC & Xeno (LIZ)	intype IC (TAMRA) & Xeno
Category	Reference Strain	Milk					
LOD dilution level (2 of 3)	1	5	5	6	5	5	5
	2	7	8	7	7	7	7
	3	6	7	6	6	6	6
R^2^ value	1	0.999	0.994	0.977	0.999	0.992	0.990
	2	0.998	1.000	0.990	0.999	0.998	0.999
	3	0.999	0.999	1.000	0.999	0.994	0.999
Percentage PCR efficiency	1	99.4	90.2	124.5	100.3	133.7	99.2
	2	106.5	105.2	110.0	95.4	115.4	94.5
	3	101.2	106.2	99.2	91.2	118.0	93.0

**Table 3:** Influenza A virus (IAV) limit of detection (LOD), coefficient of determination (R^2^), and percentage PCR efficiency for AgPath-ID/Xeno and IndiMix JOE FRV/intype IC assays in milk and semen.

RT-PCR Chemistry		AgPath-ID	IndiMix JOE FRV	AgPath-ID	IndiMix JOE FRV
Internal Control		Xeno	intype IC	Xeno	intype IC
Category	Reference Strain	Milk		Semen	
LOD dilution level (2 of 3)	1	5	5	5	5
	2	6	6	6	7
	3	6	6	7	7
R^2^ value (range)	1	0.994–0.998	0.996–0.999	0.990–0.997	0.995–0.997
	2	1.000–1.000	0.999–0.999	0.998–0.999	0.998–0.999
	3	0.998–1.000	0.998–0.999	0.995–0.999	0.997–0.998
Percentage PCR Efficiency (range)	1	90.2–103.5	96.5–101.9	110.6–118.0	116.3–118.9
	2	97.9–105.2	89.3–95.4	98.5–109.0	98.5–114.6
	3	95.3–106.2	90.2–95.8	100.6–120.7	100.6–116.9

## Data Availability

The original contributions presented in this study are included in the article/Supplementary Materials. Further inquiries can be directed to the corresponding author.

## Supplementary Materials

The following supporting information can be downloaded at https://www.mdpi.com/article/10.3390/pathogens15060600/s1. Supplemental Data S1. Influenza A virus (IAV) CT values for AgPath-ID with and without intype IC and IndiMix JOE with intype IC using the NAHLN and the FRV protocols with avian IAV-spiked serial diluted samples. Supplemental Data S2. Precision of avian influenza A virus (IAV) at high and low concentrations with AgPath-ID and IndiMix JOE with intype IC. Supplemental Data S3. Influenza A virus (IAV), Xeno, and intype IC CT values using six different assay combinations with IAV-spiked serial diluted milk samples. Supplemental Data S4. CT values for AgPath-ID with Xeno and IndiMix JOE with intype IC using the NAHLN and FRV protocols with IAV-spiked serial diluted milk and semen samples. Supplemental Data S5. Xeno and intype IC CT values from AgPath-ID with Xeno and IndiMix JOE with intype IC using the NAHLN and FRV protocols with IAV-spiked serial diluted avian, milk and semen samples. Supplemental Data S6. Influenza A virus (IAV) CT values from AgPath-ID with or without Xeno and IndiMix JOE with intype IC using the NAHLN and FRV protocols with positive and negative avian, milk, and semen samples. Supplemental Data S7. Xeno and intype IC CT values from AgPath-ID with or without an internal control and IndiMix JOE FRV with intype IC using the NAHLN and FRV protocols with positive and negative avian, milk, and semen samples. Supplemental Data S8. Influenza A virus (IAV), Xeno, and intype IC CT values with known status of IAV milk samples tested at Wisconsin Veterinary Diagnostic Laboratory (WVDL) and Ohio Department of Agriculture, Animal Disease Disgnostic Laboratory (ODA).

## Abbreviations

The following abbreviations are used in this manuscript.

AgPath-ID	AgPath-ID One-Step RT-PCR Reagents
ANOVA	analysis of variance
CT	cycle threshold
CV	coefficient of variation
FRV	fast thermocycling and reduced volume
HPAI	highly pathogenic avian influenza
IAV	influenza A virus
IC	internal control
intype IC	intype IC-RNA
LOD	limit of detection
LPAI	low-pathogenicity avian influenza
NAHLN	National Animal Health Laboratory Network
ODA	Ohio Department of Agriculture, Animal Disease Diagnostic Laboratory
PBS	phosphate-buffered saline
RT-PCR	reat-time polymerase chain reactions
R^2^	coefficient of determination
USDA	United States Department of Agriculture
Vet-LIRN	Veterinary Laboratory Investigation and Response Network
WVDL	Wisconsin Veterinary Diagnostic Laboratory
Xeno	VetMAX Xeno Internal Positive Control RNA—VIC Assay
Xeno (LIZ)	VetMAX Xeno Internal Positive Control RNA—LIZ Assay
